# Keystone Species in Pregnancy Gingivitis: A Snapshot of Oral Microbiome During Pregnancy and Postpartum Period

**DOI:** 10.3389/fmicb.2018.02360

**Published:** 2018-10-09

**Authors:** Preethi Balan, Yap Seng Chong, Shivshankar Umashankar, Sanjay Swarup, Wong Mun Loke, Violeta Lopez, Hong Gu He, Chaminda Jayampath Seneviratne

**Affiliations:** ^1^Discipline of Oral Sciences, Faculty of Dentistry, National University of Singapore, Singapore, Singapore; ^2^Department of Obstetrics and Gynaecology, Yong Loo Lin School of Medicine, National University Hospital, Singapore, Singapore; ^3^Department of Biological Sciences, Faculty of Science, National University of Singapore, Singapore, Singapore; ^4^Singapore Centre for Environmental Life Sciences Engineering, National University of Singapore, Singapore, Singapore; ^5^Synthetic Biology for Clinical and Technological Innovation, Centre for Life Sciences, National University of Singapore, Singapore, Singapore; ^6^Alice Lee Centre for Nursing Studies, Yong Loo Lin School of Medicine, National University of Singapore, Singapore, Singapore

**Keywords:** pregnancy, microbiome, gingivitis, oral health, adverse pregnancy (birth) outcomes

## Abstract

It is well known that pregnancy is under the constant influence of hormonal, metabolic and immunological factors and this may impact the oral microbiota toward pregnancy gingivitis. However, it is still not clear how the oral microbial dysbiosis can modulate oral diseases as oral microbiome during pregnancy is very poorly characterized. In addition, the recent revelation that placental microbiome is akin to oral microbiome further potentiates the importance of oral dysbiosis in adverse pregnancy outcomes. Hence, leveraging on the 16S rRNA gene sequencing technology, we present a snapshot of the variations in the oral microbial composition with the progression of pregnancy and in the postpartum period and its association with pregnancy gingivitis. Despite the stability of oral microbial diversity during pregnancy and postpartum period, we observed that the microbiome makes a pathogenic shift during pregnancy and reverts back to a healthy microbiome during the postpartum period. Co-occurrence network analysis provided a mechanistic explanation of the pathogenicity of the microbiome during pregnancy and predicted taxa at hubs of interaction. Targeting the taxa which form the ecological guilds in the underlying microbiome would help to modulate the microbial pathogenicity during pregnancy, thereby alleviating risk for oral diseases and adverse pregnancy outcomes. Our study has also uncovered the possibility of novel species in subgingival plaque and saliva as the key players in the causation of pregnancy gingivitis. The keystone species hold the potential to open up avenues for designing microbiome modulation strategies to improve host health during pregnancy.

## Introduction

The oral cavity is one of the most clinically relevant habitats in humans. The diverse microorganisms of the oral cavity have been associated with numerous oral and systemic diseases (He et al., 2015; Simpson and Thomas, 2016). Currently, the expanded Human Oral Microbiome Database (eHOMD) contains a total of 770 microbial species, which includes 687 species from version 14.51 of HOMD and 83 species have been added based on publicly available data on the microbiota of the aerodigestive tract outside of the mouth (Chen et al., 2010; Dewhirst et al., 2010; Verma et al., 2018) The diversity and richness of the oral microbial community can be modified by fluctuations in the oral environment that may occur as a consequence of the systemic changes in the human body (Avila et al., 2009). Pregnancy represents one such altered environment where the body undergoes hormonal, metabolic, and immunological changes (Kumar and Magon, 2012). A dramatic rise in the level of sex hormones like progesterone and estrogens and altered immune responses during pregnancy can cause a significant impact on the human oral microbiome (Kumar and Magon, 2012; Nuriel-Ohayon et al., 2016).

Changes in the oral microbiota and the oral ecosystem may predispose the pregnant women to a higher risk of developing “gingival diseases” or exacerbated gingival inflammation. As a result, pregnancy gingivitis has been reported to be the most common oral manifestation during pregnancy with a prevalence of 35–100%, depending on the study cohort (Onigbinde et al., 2014). Moreover, gingival inflammation is also considered to be a potential risk factor for developing adverse pregnancy outcomes (Han, 2011). Interestingly, a recent sequencing-based study has shown that the microbial taxa detected in the placenta are akin to the commensal species in the oral microbiome rather than microbes derived from the urogenital tract (Aagaard et al., 2014). Hence, it is likely that the oral microbiome plays an important role in the health and disease status of the pregnant mother as well as the developing fetus.

Previous studies using culture-based and PCR-based methods have identified the association of anaerobic species such as *Porphyromonas gingivalis, Prevotella intermedia, Treponema denticola, Tannerella forsythia, Campylobacter rectus, Prevotella nigrescens*, and *Treponema denticola* with gingival bleeding during pregnancy (Kornman and Loesche, 1980; Jensen et al., 1981; Gursoy et al., 2009; Carrillo-de-Albornoz et al., 2010). However, this knowledge has been challenged by current studies based on DNA-sequencing platforms. Apparently, only one prospective study on the oral microbiome of pregnant women has been reported in the literature and none in the Chinese population (DiGiulio et al., 2015). Therefore, taking this research gap into consideration, the present study examined the changes in the oral microbiome that occur during pregnancy and the postpartum period of the Chinese population in Singapore. Both saliva and subgingival samples were collected from 24 patients across the three trimesters of pregnancy and during the postpartum period. Herein, we present a snapshot of the variations in the oral microbial composition and diversity with the progression of pregnancy and in the postpartum period, leveraging on the 16S rRNA gene sequencing technology.

## Materials and Methods

### Cohort Design

Twenty-four participants were enrolled in this study through the prenatal clinic of a tertiary public hospital in Singapore. The study was approved by the National Health Group Domain Specific Review Boards (NHG DSRB Ref: 2014/00979). The inclusion criteria for the study were Singaporean Chinese pregnant women aged 21 years or older, with a gestational age of ≤12 weeks for the first trimester; 21–24 weeks for the second trimester; and 32–36 weeks for the third trimester; and 6 weeks for the postpartum analysis. The exclusion criteria were non-Chinese pregnant women and Chinese pregnant women with known systemic diseases and smoking habit; participants who have visited the dental clinic in the past 6 months for any gingival complaints; and prior gingival bleeding before the start of pregnancy. The study comprised 10 participants each across the three trimesters of pregnancy and 10 participants in the postpartum period, from whom a total of 40 saliva samples and 40 SGP samples were collected. Of these 40 participants, none in the first trimester attended any further follow-up sampling; six women in the second trimester attended sampling until the postpartum period; nine women in the second trimester attended sampling till the third trimester and seven women in the third trimester attended sampling till the postpartum period. The recruitment algorithm is represented in **Supplementary Figure S1**. A self-reported questionnaire was employed to obtain participants’ demographic data and assess their oral health characteristics.

### Clinical Assessment and Sample Collection

The clinical assessment of gingival bleeding was performed using the GBI of Ainamo and Bay (1975). A blunt periodontal probe was passed along the gingival crevice at the labial and lingual surfaces of the teeth. If bleeding occurred within 10–15 s, a positive score of “1” was given while a negative score of “0” was given for surfaces with no bleeding. The number of elicited bleeding points was totaled and divided by the number of gingival margins examined. The plaque index was graded on a scale of 1–3, where a score of 1 was considered “good” when dental plaque covered less than one third of all teeth present, score of 2 was considered “average” when dental plaque covered more than one third of the teeth but less than half of all teeth present, and a score of 3 was considered “poor” when half or more than half of all teeth present had dental plaque/calculus.

Unstimulated saliva was collected in a sterile, disposable 15 ml tube after thorough rinsing of the mouth. Samples of SGP were collected from all teeth between the incisors and the first molars in each of the four quadrants by inserting sterile paper points for 15 s. The plaque samples collected on the paper points were then suspended in a 2 ml tube containing 200 μL of TE buffer solution (pH 8.0). The saliva and SGP samples were frozen instantaneously on ice, transported, and stored at -80°C until further analysis. All samples used in this study were collected by the same clinician and the same protocol was used to limit sampling variability.

### DNA Extraction, Amplicon Synthesis, and Sequencing

Saliva and SGP samples were centrifuged at 8000 rpm for 10 min at 4°C to collect the cell pellet containing total DNA. Supernatant was separated from the cell pellet and DNA was extracted from the pellet using QiAamp UCP Pathogen Mini-kit (Qiagen Inc., Valencia, CA, United States) according to the manufacturer’s instructions. A quantitative 16S rRNA PCR was performed to determine the amount of bacterial template DNA in the samples and a bacterial 16S rRNA amplicon library spanning the variable region V4 was generated. The DNA sequences of the amplicon library were determined using Illumina MiSeq technology at the Beijing Genome Institute [(BGI Tech Solutions (Hongkong) Co., Ltd., Hongkong].

### Sequence Analysis

Paired-end reads were generated using the Illumina MiSeq platform. The tags were clustered into OTUs with a 97% threshold using UPARSE, and chimeras were filtered out by using UCHIME (v4.2.40) to form OTU representative sequences, with singleton OTUs excluded. Sequences representative of OTUs were taxonomically classified using the RDP Classifier v.2.2 against the Greengenes database, using 0.8 confidence value as cut-off. The representative sequences were aligned against the Silva core set (Silva_108_core_aligned_seqs) using PyNAST by ‘align_seqs.py’. Alpha-diversity indices were estimated based on Observed species, Chao1, Shannon, and Simpson values (Shannon, 1948; Chao, 1984), which reflect the species diversity of the community, and are affected by both species richness and species evenness. These indices were calculated using Mothur (v1.31.2) and the corresponding rarefaction curves were drawn by R (v3.0.3). Fishers alpha diversity was calculated using fisher.alpha function of ‘vegan’ package (Oksanen et al., 2013), while the good coverage was calculated using the goods function of ‘QsrUtils’ package, and the boxplot was plotted using the ‘ggplot2’ package in R (Wickham, 2018). UniFrac-based PCoA was performed using QIIME. Metastats and R (v3.0.3) were used to determine differentially abundant taxonomic groups.

### Statistical Analysis

Paired *t*-tests were performed to compare the alpha diversity among the trimester groups and with the postpartum group. PERMANOVA with 999 permutations was performed on the unweighted UniFrac distances using the *adonis* function in ‘vegan’ package in R (Oksanen et al., 2013). The distance-based tests of homogeneity of multivariate dispersions, to evaluate differences in structure centroid and dispersion (beta diversity), were performed using both the ANOVA and permutation test for homogeneity. Wilcoxon rank sum test was used to compare taxonomic abundance in the cross-sectional analysis. Spearman correlation was used to obtain pairwise score and to construct microbial interaction network models for pregnant and postpartum subjects. Pairwise network comparisons were carried out at global and module levels. Cytoscape ‘Network Analyzer’ plugin (Shannon et al., 2003) was used to characterize the global topological properties of networks during the three trimesters of pregnancy and the postpartum period. For module level comparisons, Molecular Complex Detection (MCODE) (Bader and Hogue, 2003) was used to extract the top highly connected modules in both pregnant and the postpartum groups. Spearman correlation coefficients were used to assess the bacterial association with GBI. Identification of keystone species was done using the CytoHubba plugin (Chin et al., 2014), which provided topological analysis by Degree, Edge Percolated Component, Maximum Neighborhood Component, Density of Maximum Neighborhood Component, and Maximal Clique Centrality (MCC) and six centralities (Bottleneck, EcCentricity, Closeness, Radiality, Betweenness, and Stress) based on the shortest paths. To assess the predictive power of the keystone species in identifying pregnancy gingivitis, a receiver operating characteristic (ROC) curve was constructed with true positive rate (Sensitivity) as a function of the false positive rate (1-Specificity) using XLSTAT add-on.

## Results

### Participant Characteristics

The mean age of the participants in the study was 31.9 (SD ± 5.4) years and the mean gestational age was 8.9 weeks (SD ± 3.3), 21.7 weeks (SD ± 1.4), and 31.6 weeks (SD ± 1.5) in the first, second, and third trimesters respectively. All participants were reported to be non-smokers. Majority of the participants (83.3%) brushed their teeth twice a day. A good Plaque Index score was obtained for 66.7% of participants, while the remaining 33.3% obtained an average plaque score. Demographic and baseline clinical characteristics of the study population have been detailed in **Supplementary Table S1**. A total number of 3,052,703 reads were obtained from the 80 saliva and SGP samples with an average of 38,158 reads per sample and an average length of 252 bp. Of these, 2,787,452 sequences were filtered from the dataset, aligned with the Greengenes database, and clustered to 736 OTUs, which were non-singletons at 97% sequence similarity. OTUs were classified into a total of 12 phyla, 65 genera, and 131 species.

### Oral Microbiome Remains Stable During the Course of Pregnancy

**FIGURE 1:**
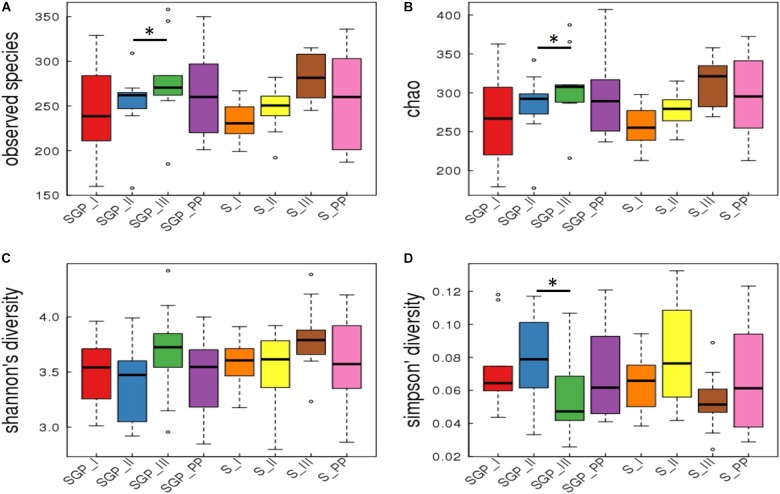
Alpha diversity indices in subgingival plaque and saliva samples. Cross sectional analysis of alpha diversity measures in SGP and saliva samples across pregnancy and postpartum period. The indices are plotted with four alpha diversity indicators; **(A)** Observed species; **(B)** Chao1; **(C)** Shannon diversity; **(D)** Simpson’s diversity. SGP, Subgingival plaque; S, Saliva; I, II, III indicate 3 trimesters of pregnancy; PP, Postpartum (^∗^*p* < 0.05).

Good’s coverage is an estimator of the proportion of non-singleton OTUs in the dataset as a measure of overall OTU sampling completion and was used to assess the adequacy of sampling. The Good’s coverage of bacteria reached 99.8% in our dataset with majority of microbial diversity being captured. Alpha diversity analysis of the forty samples of SGP showed that three of the alpha diversity measures (Observed species, Chao 1, and Simpson index) were significantly different for only SGP samples between the second and third trimesters (**Figure 1**). Fisher’s alpha diversity, which is an indicator for logarithmic changes in relative abundances corroborated these findings and has been represented in **Supplementary Figure S2** On further analysis, it was observed that the significant differences in taxa arose due to inter-patient differences, which accounted for nearly 76% of the total microbial variation (**Supplementary Table S2**). Hence, analysis was performed among the longitudinal samples of six participants, who completed the sampling from the second trimester until the postpartum period. The longitudinal comparison showed that, in SGP samples, the observed number of species and Chao_1 index was significantly higher in the third trimester compared to the second trimester (**Supplementary Figure S3**). However, the Shannon and Simpson diversity index, which is a mathematical measure of diversity in a community, did not show any significant difference across the pregnancy trimesters and postpartum period. The saliva samples did not show any significant trend in any of the alpha diversity indices in both cross sectional and longitudinal analysis. These results indicate that the alpha diversity, in both inter-individual and intra-individual samples, remains stable across the pregnancy trimesters and postpartum period.

**FIGURE 2:**
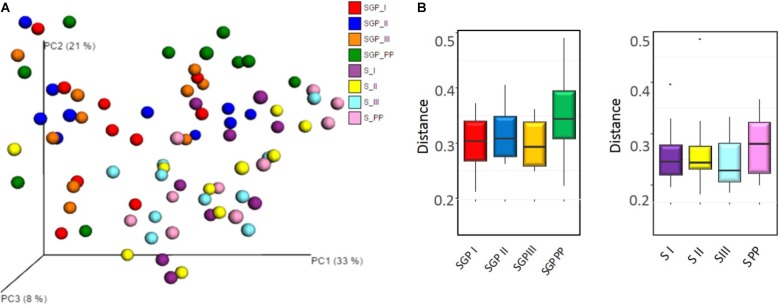
Community structure analysis of sub gingival plaque and saliva samples **(A)** Principal Coordinate analysis (PCoA) based on weighted UniFrac distance. No distinct grouping of the bacterial communities across any trimesters of pregnancy and the postpartum period in saliva and SGP samples. **(B)** Boxplot showing the distance-based tests of homogeneity of multivariate dispersions. No significant differences in the distances of group members to the group centroid was observed. SGP, Subgingival plaque; S, Saliva; I, II, III indicate 3 trimesters of pregnancy; PP, Postpartum.

Beta diversity analysis demonstrated a distinct separation between saliva and SGP samples. However, the PCoA ordination did not reveal any distinct grouping of the bacterial communities across any of the trimesters of pregnancy or the postpartum period from both the niches (*p* = 0.209) (**Figure 2A**). In addition, distance-based tests of homogeneity of multivariate dispersions, also did not show any significant difference between trimesters (**Figure 2B**). Taken together, these results suggest that the oral microbial community structure is closely related and does not dramatically remodel in its diversity with the progression of pregnancy.

### Oral Microbiome Is Pathogenic During Pregnancy and Reverts to Healthy During Postpartum Period

The bacterial community of SGP and saliva was examined at different taxonomic levels. At the phylum level, the microbial composition during pregnancy was dominated by members of Firmicutes (>30%), Bacteroidetes (>20%), and Actinobacteria (>5%) in both saliva and SGP samples (**Supplementary Figure S5A**). At the genus level, the genera Prevotella (15.65%), Fusobacterium (15.63%), Streptococcus (13.30%), Veillonella (6.80%) and Terrahaemophilus (5.01%) were most abundant in SGP and collectively represented more than 50% of sequences identified in SGP (**Supplementary Figures S4, S5B**). In saliva, the genera Prevotella (21.8%), Streptococcus (17.74%), Veillonella (10.17%), Neisseria (9.50%), and Terrahaemophilus (7.60%), along with some unclassified genera (6.92%) were detected at more than 5% abundance and comprised over 70% of all detected sequences in saliva.

**FIGURE 3:**
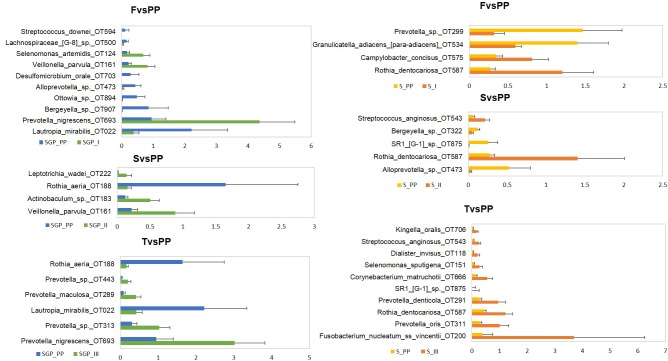
Differential abundance of subgingival and saliva samples at species level. F, First trimester; S, Second trimester; T, Third trimester; PP, Postpartum (*p* < 0.05).

At species level, *unclassified species, Streptococcus sp. OT 058* and *Terrahaemophilus aromaticivorans* formed the highest abundance in both saliva and SGP samples during pregnancy (**Supplementary Figure S5C**). The pathogenic species *Prevotella nigrescens, Prevotella oris* and *Porphyromonas gingivalis* were most abundant in SGP, while *Prevotella_sp._oral_taxon_313* and *Prevotella melaninogenica* were most abundant in saliva. *Fusobacterium_nucleatum ss vincentii* showed higher abundance in SGP (13–15%) as compared to saliva (<4%) samples. Comparison of species with relative abundance that surpassed the 0.1% threshold in SGP showed that there was a significant drop in the abundance levels of pathogenic species—*Veillonella parvula*, *Prevotella species* and *Actinobaculum species* (*p* < 0.05) in the postpartum period compared with the three trimesters of pregnancy (**Figure 3**). Similarly, in the saliva samples, the pathogenic species *Fusobacteium nucleatum vincentii, Dialister invisus, Prevotella oris, Prevotella denticola, Cornybacterium matruchotii, Rothia dentocariosa, Streptococcus anginosus, Selenomonas sputigena*, and *Kingella orali* s were decreased significantly (*p* < 0.05) in the postpartum period than the pregnant condition. Conversely, bacterial species associated with health such as *Lautropia mirabilis*, *Rothia aeria, Granulicatella adiacens* and *SR1 species* repopulated in the oral microbiome during the postpartum period. It is likely that the pathogenic bacterial species in the oral microbiota thrive during the course of pregnancy, but their abundance declines during the postpartum period with the re-establishment of healthy oral microbiota.

### Network Analysis

To compare the ecological relationships across the bacterial communities, correlation based networks were generated for the pregnancy group (by merging all three trimesters) and for the postpartum group. The networks were then analyzed at the global and module levels. The topological properties at the global level of the two networks are shown in **Table 1**. The topological properties showed that the network heterogeneity was more during pregnancy than postpartum period (1.26 vs. 0.59), while the network density was low during pregnancy (0.029) as compared to the postpartum period (0.128) (**Table 1**). There were 53 connected components in the pregnant group, while only 5 components were detected in the postpartum group. The average number of neighbors were observed to be higher in postpartum period (33.66) than pregnancy (7.66). These topological network properties of the pregnant and postpartum group suggest that the microbial community during pregnancy tend to have natural divisions within them which were not well connected. In contrast, the microbial community during the postpartum period was more coherent with strong inter-dependency within the members of the group. Thus it can be deduced that the microbial communities in the pregnant and postpartum conditions have different co-occurrences and mutual exclusion relationships.

**Table 1:** Topological properties of interaction networks.

Network property	Pregnant group	Postpartum group
Nodes	264	264
Edges	1012	4444
Edge to node ratio	3.8:1	16.8:1
Clustering coefficient	0.300	0.464
Connected components	53	5
Network diameter	11	5
Network centralization	0.124	0.181
Shortest path	40240 (57%)	67340 (96%)
Characteristic path length	4.045	2.212
Average number of neighbors	7.667	33.667
Network density	0.029	0.128
Network heterogeneity	1.264	0.593
Isolated nodes	46	4


**FIGURE 4:**
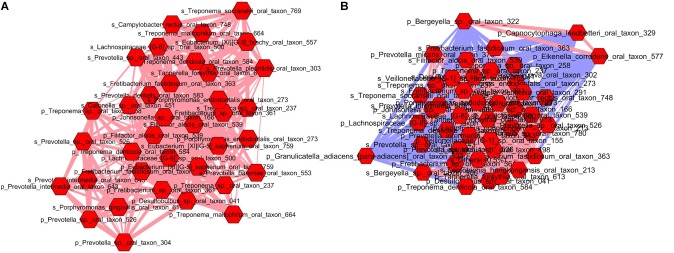
Top module in pregnant **(A)** and postpartum **(B)** subgingival and salivary microbiome. Each node represents a bacterial species and each edge represents a co-occurrence/co-exclusion relationship. Nodes are colored based on first neighbors of *Filifactor alocis* sp. in both saliva and subgingival plaque. Edge width is proportional to the correlation coefficient and color indicates the sign of the association (blue: negative, pink: positive). Prefix ‘p’ and ‘s’ denote species from subgingival plaque and saliva respectively.

To gain deeper insights into these differences, microbial communities were analyzed at the module level. As a result, we uncovered 8 modules (cluster) in the pregnancy group (**Supplementary Figure S6**) and 11 modules in the postpartum group (**Supplementary Figure S7**). **Supplementary Tables S3, S4** lists the results of MCODE cluster-detection in the two bacterial interaction networks. Interestingly, in the pregnant group, there was only a single strong cluster (score 20.51) with 36 nodes and 359 edges, while the other clusters were small with 3–7 nodes and 3–16 edges. The postpartum network also showed a strong cluster (score 40.83) with 44 nodes and 878 edges. However, unlike the pregnant group network, the postpartum network demonstrated additional 5 large but moderately strong clusters with 21–36 nodes and 47–180 edges. This indicates that oral microbiome during pregnancy is dominated by a single cluster of taxa, while the postpartum period promotes development of other clusters of taxa with strong interdependencies. Due to space constraints, we limited our analysis to the top module in each of pregnant and postpartum network. The top modules in both pregnant (**Figure 4A**) and postpartum group (**Figure 4B**) were dominated by pathogenic species which have been implicated in gingival or periodontal diseases. When the networks were connected by their first neighbors (direct correlations), we observed that *Filifactor alocis* formed the hub OTU around which maximum associations were centered in both the pregnant and postpartum networks. Interestingly, in the top module of pregnant group, the nodes were linked with only positive edges, suggestive of a synergistic relationship between the pathogenic species. In contrast, the positive interactions in the postpartum microbial network was also accompanied by negative interactions by health associated species such as *Bergeyella sp OT 322, Capnocytophaga leadbetteri OT 329, Eikenella corrodens OT 577*, and *Granulicatella_adiacens para-adiacens OT 534*. This invasion of negative interactions by health-associated taxa into the cluster of pathogenic species during the postpartum period explains partly how the microbiome restores to health after pregnancy.

### Oral Microbial Keystone Species Associated With Pregnancy Gingivitis

Deciphering microbial interactions and detecting the keystone species associated with disease are of great value for understanding the diseases pathologies. Hence, to identify the keystone species associated with pregnancy gingivitis, firstly, the level of pregnancy gingivitis was analyzed during each trimester of pregnancy and in the postpartum period using Ainamo GBI. The gingival bleeding was observed to increase from the first to the second trimester and decreased thereafter toward the postpartum period (**Supplementary Table S1**). Pairwise Spearman correlation analysis was then performed to assess whether the GBI correlated with specific bacterial groups in saliva and SGP. The Spearman correlation results showed that 31 species from SGP and 27 species from saliva significantly correlated with the GBI (defined as FDR < 0.200) (**Supplementary Figure S8**). Notably, the identified species that were present in more than 0.5% abundance included the periopathogens such as *Porphyromonas gingivalis* (2.22%), *Treponema denticola* (1.10%) and *Fretibacterium* sp. *OT 361* (0.67%) in SGP samples and *Prevotella_intermedia* (0.56%) in the saliva samples. In addition, *Porphyromonas endodontalis* was also observed in both SGP (0.89%) and saliva (0.91%) in high abundance. Intriguingly, the majority of the species associated with gingival bleeding were present in low abundance indicating the possibility of novel phylotypes being associated with pregnancy gingivitis, an observation that has not been reported before.

**FIGURE 5:**
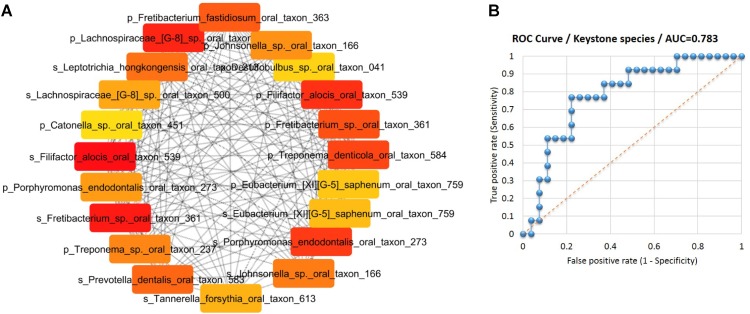
Identification of keystone species for pregnancy gingivitis. **(A)** Top 20 nodes (species) ranked by Maximal Clique Centrality and displayed in circular layout. Nodes colored based on rank; dark color denotes high ranks **(B)** ROC curves of the keystone species in the identification of pregnancy gingivitis based on its relative abundance in saliva or subgingival plaque. (AUC denotes areas under the ROC curve).

Subsequently, an interaction network of the species associated with gingival bleeding during pregnancy was constructed based on Spearman’s correlation of the 31 species from SGP and 27 species from saliva. To identify the hubs or keystone species in the network, the nodes (species) were ranked by MCC algorithm (Chin et al., 2014) into top 20 species (**Figure 5A**). The species ranking were further substantiated by other algorithms of CytoHubba plugin (**Supplementary Data Sheet S3**). Thus, 11 SGP species and 9 saliva species were identified as the keystone players in the pathogenesis of pregnancy gingivitis.

To determine the diagnostic values of keystone species in pregnancy gingivitis, ROC curve was constructed. The area under the ROC curve (AUC) of the key species group was 0.783, indicating a good diagnostic performance (**Figure 5B**). The cut-off points of the key species group were determined at 5.28% (*p* = 0.001, sensitivity 0.76, specificity 0.77). These results indicate that women who have an abundance threshold of keystone species over 5.28% in SGP and saliva pose a high risk of developing gingivitis during pregnancy.

## Discussion

The high prevalence of gingival bleeding among women during pregnancy, could be due to the microbial perturbations influenced by immune modulations and hormonal changes (Mealey and Rose, 2008; Wu et al., 2015). Despite having a significant clinical relevance, high-resolution data on the oral microbiome during pregnancy and postpartum period are scarcely available. Therefore, using 80 samples of SGP and saliva, we analyzed the structure and composition of microbial community during pregnancy and identified the bacterial associations within the community that predispose women to pregnancy gingivitis.

Firstly, we observed that the inter-patient differences accounted for nearly 76% of the total microbial variation and was the predominant source of variation in the dataset. After offsetting the inter-patient variability, it was observed that species richness and diversity of the SGP and saliva samples were relatively stable during the course of pregnancy. Beta diversity analysis also showed that the differences in microbiome of patients is independent of the trimester and is more of an innate property of the patients themselves. These findings were similar to that of a previous study, which demonstrated that the temporal and spatial variations in the oral microbiota tends to remain stable over gestational time (DiGiulio et al., 2015).

Despite the stability of the oral microbiome during pregnancy, it was intriguing to observe the dominance of pathogenic bacterial community during pregnancy, which attempts to re-establish into a healthy community during the postpartum period. We observed that the abundance of pathogenic taxa from genera *Prevotella, Streptococcus* and *Veillonella* in both SGP and saliva samples were more during pregnancy. This finding was in accordance with the recent study on oral microbiome in a similar population of pregnant Chinese women (Lin et al., 2018). However, the abundance of taxa observed in our study was higher than this study due to the discrepancies in sampling habitats. In contrast to the pregnant state, the genera most abundant in healthy non-pregnant subjects have been reported to be *Haemophilus*, *Neisseria*, *Streptocoocus* and *Rothia*, while the genera *Fusobacteria* and *Spirochaetes* were reported be in less abundance (Aas et al., 2005; Bik et al., 2010). In our cohort, the genera *Fusobacteria* was more prevalent with SGP (15.63%) showing more abundance than saliva (3.82%). The increased relative abundance of pathogenic taxa was observed at the species level as well with *Prevotella species, Porphyromonas gingivalis*, and *Fusobacterium nucleatum* showing higher abundance during pregnancy. The species *Prevotella intermedia*, *Prevotella melaninogenica*, and *Porphyromonas gingivalis*, have been reported to be abundant in previous culture based studies on pregnant women and have shown association with pregnancy gingivitis (Adriaens et al., 2009; Gursoy et al., 2009; Fujiwara et al., 2017). Interestingly, we observed that there was a significant decline in the abundance of pathogenic species from pregnancy to postpartum period. The species which showed significant drop in abundance in our cohort have not been identified previously and were largely in accordance with the comprehensive description of the microbiota in gingivitis of non-pregnant subjects (Kistler et al., 2013; Huang et al., 2014). This observation furthermore supports the pathogenicity of taxa during pregnancy. The decrease in the abundance levels of pathogenic species in the postpartum period was accompanied by simultaneous repopulation of healthy microbiome such as *Lautropis mirabilis* sp., *Rothia aeria, Granulicatella adiacens and SR1 sp* (Bik et al., 2010; Segata et al., 2012; Tsuzukibashi et al., 2017). These species have been reported to be dominating in the health-associated microbial communities in healthy subjects in previous studies (Abusleme et al., 2013; Hong et al., 2015). Pregnancy-related transition to pathogenic microbiome and its restoration to health during the postpartum period could be a result of complex host–microbial interactions that may be taking place under the influence of hormonal and immunological factors (Wu et al., 2015). In addition, pregnancy-induced perturbations in the oral cavity may disrupt the ecological balance maintained by interspecies interactions (Avila et al., 2009). These may trigger the overgrowth of species with pathogenic potential and suppress the healthy microbiome (Jenkinson and Lamont, 2005).

Further, our network analysis revealed that bacterial communities have different co-occurrence and co-exclusion relationships during pregnancy and postpartum period. The microbial network during pregnancy was observed to be inhomogeneous and sparse due to the development of natural divisions in the network. Analysis at the module level identified 8 clusters in the pregnancy network, of which only one was observed to have exceptional strength, and this module included positively associated pathogenic species centered on *Filifactor alocis* *species*. This was similar to the synergistic ecological pattern involving *Filifactor alocis* observed in a study of Chinese population with periodontitis (Chen et al., 2015). The clustering of potentially pathogenic groups into distinct hubs suggests how these pathogens mutually cooperate to create an imbalance in the microbial community. This in turn may lead to the creation of a milieu that is progressively pathogenic and detrimental to health. Contrary to this, the postpartum microbial network was more cohesive and included variable modules of optimum strength reflecting strong inter-dependencies. These relationships may be crucial for maintaining good health, and disruption of these interactions may lead to instability in the ecosystem or disease. However, the strongest cluster in the postpartum period microbial network was also centered on *Filifactor alocis* similar to the pregnancy microbial network. Previous studies have reported that in healthy non-pregnant population, the largest microbial representation is by *Actinomyces* spp*., Bergeyella* sp*., Kingella oralis, Lautropia mirabilis, Rothia aeria*, and *Streptococcus* spp. *Granulicatella_adiacens, Eikenella corrodens*, and *Capnocytophaga leadbetteri* (Abusleme et al., 2013; Diaz et al., 2016). The key finding in our study was the identification of negative interactions of majority of these health-associated species with the likely pathogens, indicating the restoration of the microbiome to health during the postpartum period. Given that all the negative interactions were associated with the pathogenic taxa in the post-partum period, the absence of these negative links in the pregnancy microbial network could be a possible cause for the decline of inhibitive effects on the pathogenic taxa in the microbiome. This also explains partly the pathogenic predisposition of the oral microbiome during pregnancy.

The dominance of pathogenic taxa could be the cause for pregnancy gingivitis, which is a most common manifestation of during this period (Silk et al., 2008). Pregnancy gingivitis shows a gradual increase during the progression of pregnancy, with an apparent resolution following parturition (Loe and Silness, 1963; Gursoy et al., 2008). The trend seen in our study cohort was concurrent with this observation. We identified 31 species from SGP and 27 species from saliva that were significantly associated with pregnancy gingivitis. These findings were concurrent with the majority of the previously well-studied anaerobes which showed high abundance in the setting of pregnancy gingivitis such as *Prevotella intermedia, Porphyromonas gingivalis, Treponema forsythia, Campylobacter rectus, Fusobacterium nucleatum*, and *Actinobacillus actinomycetemcomitans* (Kornman and Loesche, 1982; Yokoyama et al., 2008; Adriaens et al., 2009; Gursoy et al., 2009; Carrillo-de-Albornoz et al., 2010). Of these, *Prevotella intermedia species* is speculated as the most abundant species in pregnancy gingivitis and its growth was found to be stimulated by direct interaction of female sex hormones on the fumarate reductase system (Kornman and Loesche, 1982; Wu et al., 2015). Contradictory to this general understanding of pathogens being abundant in disease, our microbiome data revealed that the majority of species associated with gingival bleeding were present in low abundance. Thus, high throughput sequencing platform used in our study has uncovered the possibility of novel low abundant species being key players in pregnancy gingivitis. However, their role in infections remains elusive, as they have not been cultivated yet.

As gingival diseases are polymicrobial diseases that require synergistic contribution of pathogens for disease progression (Short et al., 2014), it is comprehensible that it is not the mere presence of the pathogenic species, but rather the interactions between specific microbes that determines the course of disease. In this context, measuring the nodes of the interaction network by their network features would help to infer their biological importance in the functioning of the ecosystem. The ranking of species associated with pregnancy gingivitis using topological algorithms helped in the identification of keystone species. Interestingly, of the 20 keystone species identified, only *Porphyromonas endodontalis* and *Fretibacterium sp. OT 361* were present in more than 0.5% abundance. This finding is in line with the “keystone bacterial hypothesis” that suggests the role of low-abundance bacterial species in modulating the oral microbiome and orchestrating the disease process (Hajishengallis et al., 2012). Moreover, the low abundance species ranked more than the previously known players of pregnancy gingivitis such as *Prevotella intermedia* and *Porphyromonas gingivalis*, indicative of their presence having more biological importance in pregnancy gingivitis.

The synergistic interactions between cultivable oral pathogens have been studied previously. An *in vitro* study has shown that steroid dependent metabolism of *Prevotella intermedia* produces large amounts of fumarate, which in turn is taken up by *Campylobacter rectus* for its growth (Grenier and Mayrand, 1986). Similarly, *Porphyromonas gingivalis* can produce isobutyric acid to enhance the growth of *Treponema denticola* and utilize the succinate produced by *Treponema denticola* for its own growth (Grenier, 1992). Saito et al. (2008) have demonstrated the invasive abilities of *Porphyromonas gingivalis* strains in the presence of *Fusobacterium nucleatum*. However, these studies have studied the mutual symbiotic enhancement of microbial growth focussing two arbitrarily selected potential pathogen. Our study has identified the novel coordinated co-occurrence of 20 species present in low abundance along with the established oral pathogens. Furthermore we have evaluated the diagnostic value of this cluster using ROC curves and demonstrated that it has the potential to identify women at high-risk to develop pregnancy gingivitis.

## Conclusion

In summary, the present study provides invaluable new insights on the modulation in the oral microbiome during pregnancy and the postpartum period. Contrary to the traditional understanding, our study has identified a potential synergistic network involving the low abundant “keystone” SGP and salivary species during pregnancy gingivitis. The keystone species hold potential to open up avenues for designing microbiome modulation strategies to improve host health during pregnancy. Considering the pilot nature of the study with limited sample size, our conclusions may be an over-simplification of the disease process. In order to obtain a definitive picture of the underlying changes associated with pregnancy gingivitis, a longitudinal study is obligatory. Nevertheless, our study lends a robust foundation for future research to develop management strategies which would help to obtain a microbial community structure favoring health during pregnancy.

## Availability of Data and Material

The datasets used and/or analyzed during the current study are available from the corresponding author on reasonable request.

## Ethics Statement

The study was approved by the Institutional Review Board of the study hospital (NHG DSRB Ref: 2014/00979). Signed informed consent was obtained from all participants upon recruitment.

## Author Contributions

CS, PB, YC, WL, HH, and VL conceived and designed the study. PB helped in data acquisition. PB, SS, and SU analyzed and interpreted the data. CS, PB, WL, HH, VL, YC, SS, and SU contributed to the writing of the manuscript. All the authors read and approved the final manuscript.

## Conflict of Interest Statement

The authors declare that the research was conducted in the absence of any commercial or financial relationships that could be construed as a potential conflict of interest.

## Abbreviations

ANOVA: analysis of variance
BGI: Beijing Genome Institute
FDR: False Discovery Rate
GBI: gingival bleeding index
HMP: human microbiome project
NHG DSRB: National Health Group Domain Specific Review Boards
OTU: operational taxonomic units
PCoA: principal coordinates analysis
PERMANOVA: Permutational Multivariate Analysis of Variance Using Distance Matrices
RDP: Ribosomal Database Project
SGP: subgingival plaque
