# Supplementary material for: Keystone Species in Pregnancy Gingivitis: A Snapshot of Oral Microbiome During Pregnancy and Postpartum Period
Source: Front Microbiol. 2018 Oct 9;9:2360. doi: 10.3389/fmicb.2018.02360 (PMC6189292; doi:10.3389/fmicb.2018.02360)
Supplement: Supplementary file 1 [file Data_Sheet_1.pdf]

Figure S1 Algorithm of subject recruitment and categorisation

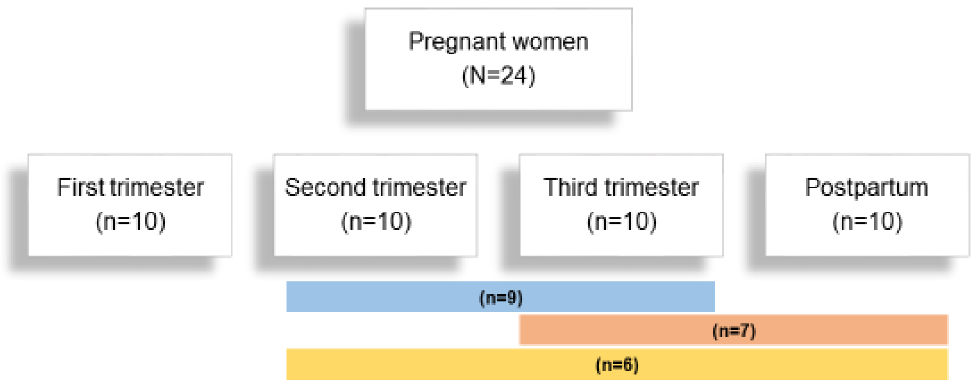

Figure S2 Fisher's alpha diversity indices in subgingival plaque and saliva samples.

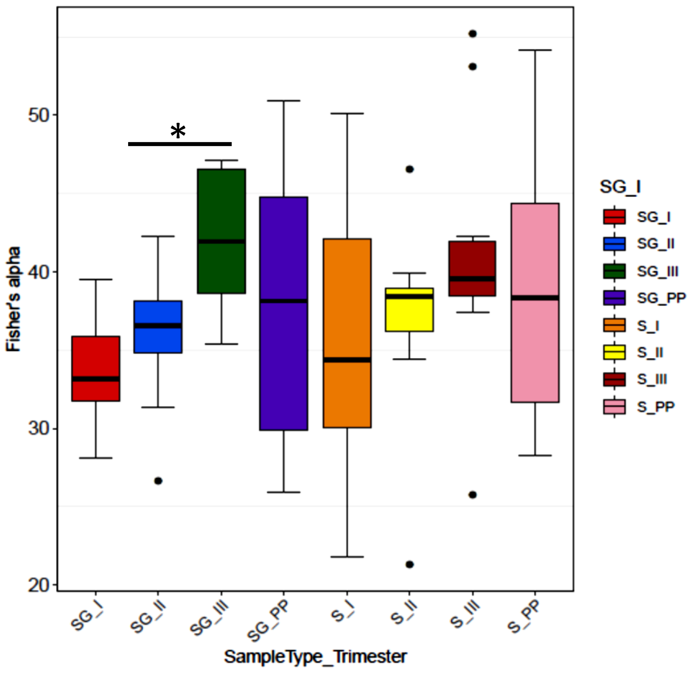

Fisher's alpha diversity estimates in the three trimesters of pregnancy and postpartum period for subgingival plaque and saliva samples. SG, Subgingival plaque; S, Saliva; I, II, III indicate 3 trimesters of pregnancy; PP, Postpartum (* $p<0.05$)

Figure S3 Alpha diversity indices in subgingival plaque and saliva samples.

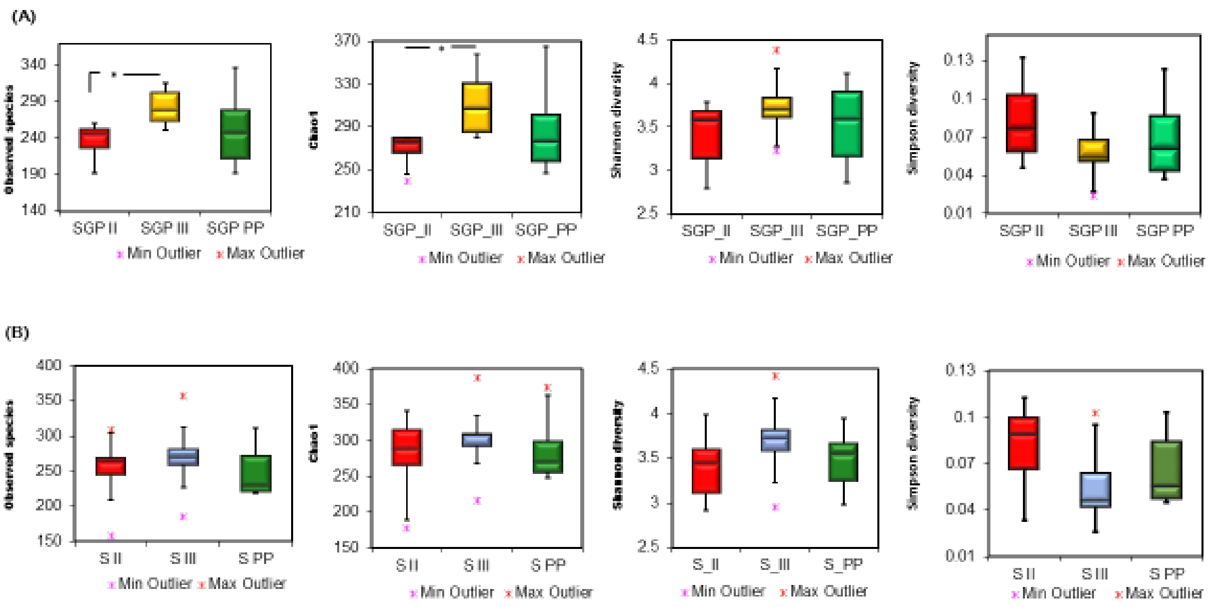

(A) Longitudinal analysis of alpha diversity measures in SGP samples from second trimester until postpartum period. **(B)** Longitudinal analysis of alpha diversity measures in saliva samples from second trimester until postpartum period. The indices plotted with four alpha diversity indicators; Observed species; Chao1; Shannon diversity. SGP, Subgingival plaque; S, Saliva; I, II, III indicate 3 trimesters of pregnancy; PP, Postpartum (* p<0.05)

Figure S4 Species clustering heat map at the level of genera.

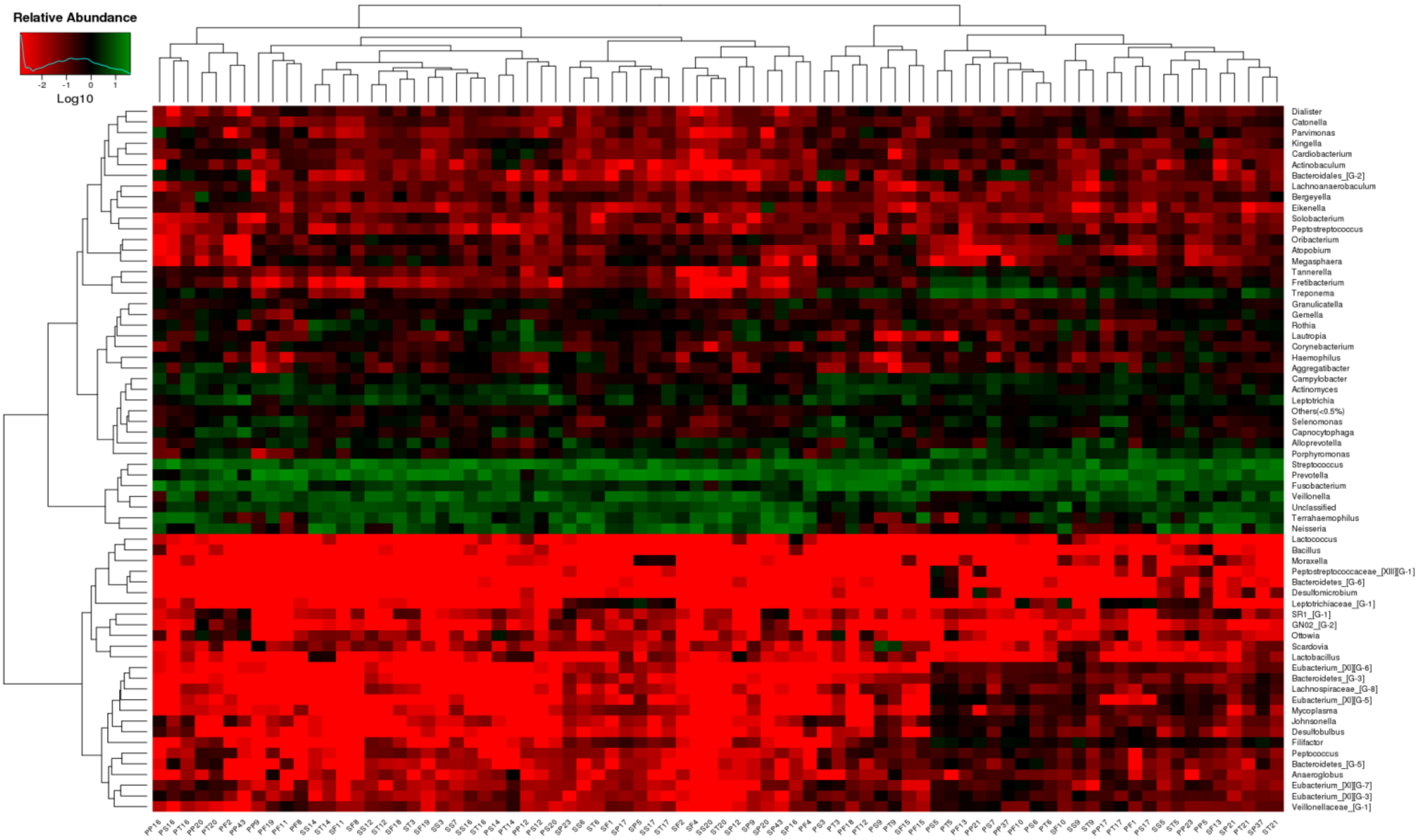

Heat map shows species clustering analysis based on the relative abundance of each genera in each sample. To minimize the differences in degree of the relative abundance value, the values were log transformed. The genera with abundance less than 0.5% in all samples were classified into 'others'

Figure S5.Taxonomic composition of subgingival and saliva samples at Phylum (A) Genus (B) and Species (C) level.

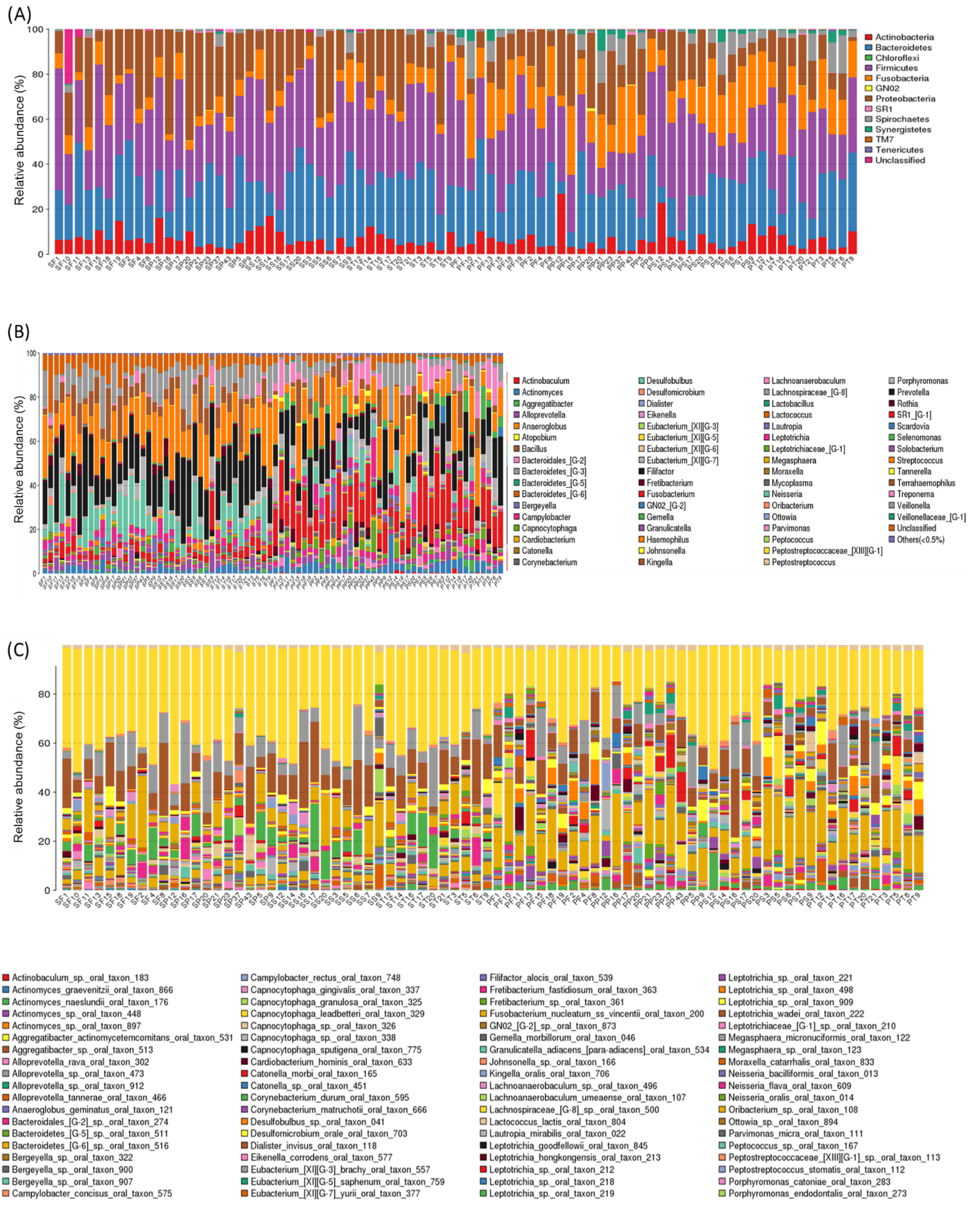

Porphyromonas_gingivalis_oral_taxon_619
Porphyromonas_sp._oral_taxon_279
Prevotella_baroniae_oral_taxon_553
Prevotella_dentalis_oral_taxon_583
Prevotella_denticola_oral_taxon_291
Prevotella_intermedia_oral_taxon_643
Prevotella_maculosa_oral_taxon_289
Prevotella_melaninogenica_oral_taxon_469
Prevotella_micans_oral_taxon_378
Prevotella_nigrescens_oral_taxon_693
Prevotella_oris_oral_taxon_311
Prevotella_oulorum_oral_taxon_288
Prevotella_pallens_oral_taxon_714
Prevotella_pleuritidis_oral_taxon_303
Prevotella_saccharolytica_oral_taxon_781
Prevotella_salivae_oral_taxon_307
Prevotella_sp._oral_taxon_292
Prevotella_sp._oral_taxon_299
Prevotella_sp._oral_taxon_304
Prevotella_sp._oral_taxon_313
Prevotella_sp._oral_taxon_317
Prevotella_sp._oral_taxon_443
Prevotella_sp._oral_taxon_472
Prevotella_sp._oral_taxon_526
Rothia_aeria_oral_taxon_188
Rothia_dentocariosa_oral_taxon_587
SR1_[G-1]_sp._oral_taxon_345
SR1_[G-1]_sp._oral_taxon_875
Scardovia_wiggsiae_oral_taxon_195
Selenomonas_artemidis_oral_taxon_124
Selenomonas_sp._oral_taxon_478
Selenomonas_sputigena_oral_taxon_151
Solobacterium_moorei_oral_taxon_678
Streptococcus_anginosus_oral_taxon_543
Streptococcus_downei_oral_taxon_594
Streptococcus_mutans_oral_taxon_686
Streptococcus_parasanguinis_II_oral_taxon_411
Streptococcus_salivarius_oral_taxon_755
Streptococcus_sp._oral_taxon_058
Tannerella_forsythia_oral_taxon_613
Terrahaemophilus_aromaticivorans_oral_taxon_826
Treponema_denticola_oral_taxon_584
Treponema_lecithinolyticum_oral_taxon_653
Treponema_maltophilum_oral_taxon_664
Treponema_socranskii_oral_taxon_769
Treponema_sp._oral_taxon_237
Treponema_sp._oral_taxon_239
Treponema_sp._oral_taxon_258
Treponema_sp._oral_taxon_263
Treponema_sp._oral_taxon_508
Veillonella_parvula_oral_taxon_161
Veillonella_sp._oral_taxon_780
Veillonella_sp._oral_taxon_917
Veillonellaceae_[G-1]_sp._oral_taxon_155
Unclassified
Others(<0.5%)

Fig. S6 Co-occurrence and co-exclusion network among species of saliva and subgingival plaque during pregnancy.

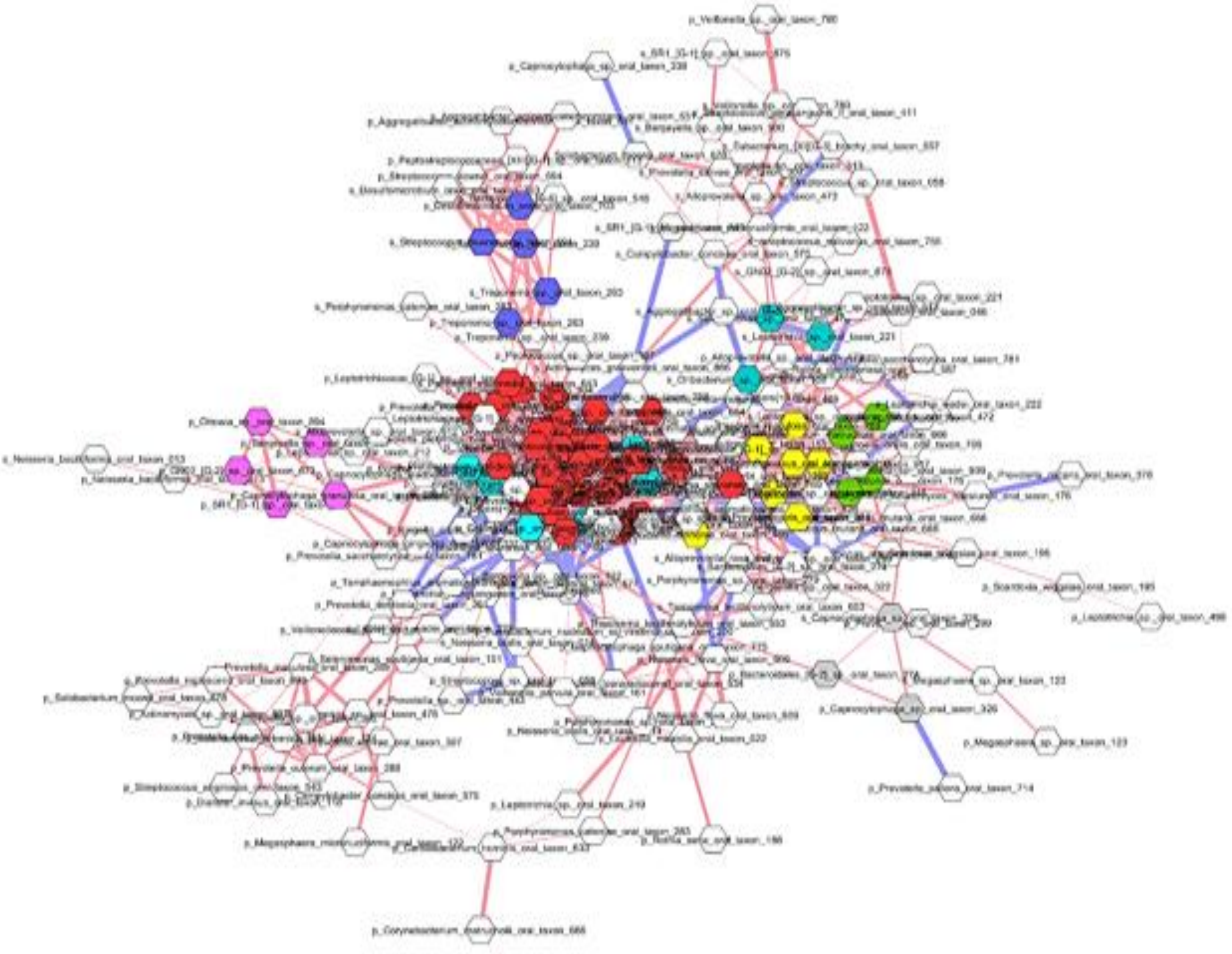

Each node represents a bacterial species and each edge represents a co-occurrence/co-exclusion relationship. Nodes are coloured based on modules which are highly connected. Edge width is proportional to the correlation coefficient and colour indicates the sign of the association (blue: negative, pink: positive).

Fig. S7 Co-occurrence and co-exclusion network among species of saliva and subgingival plaque during postpartum period.

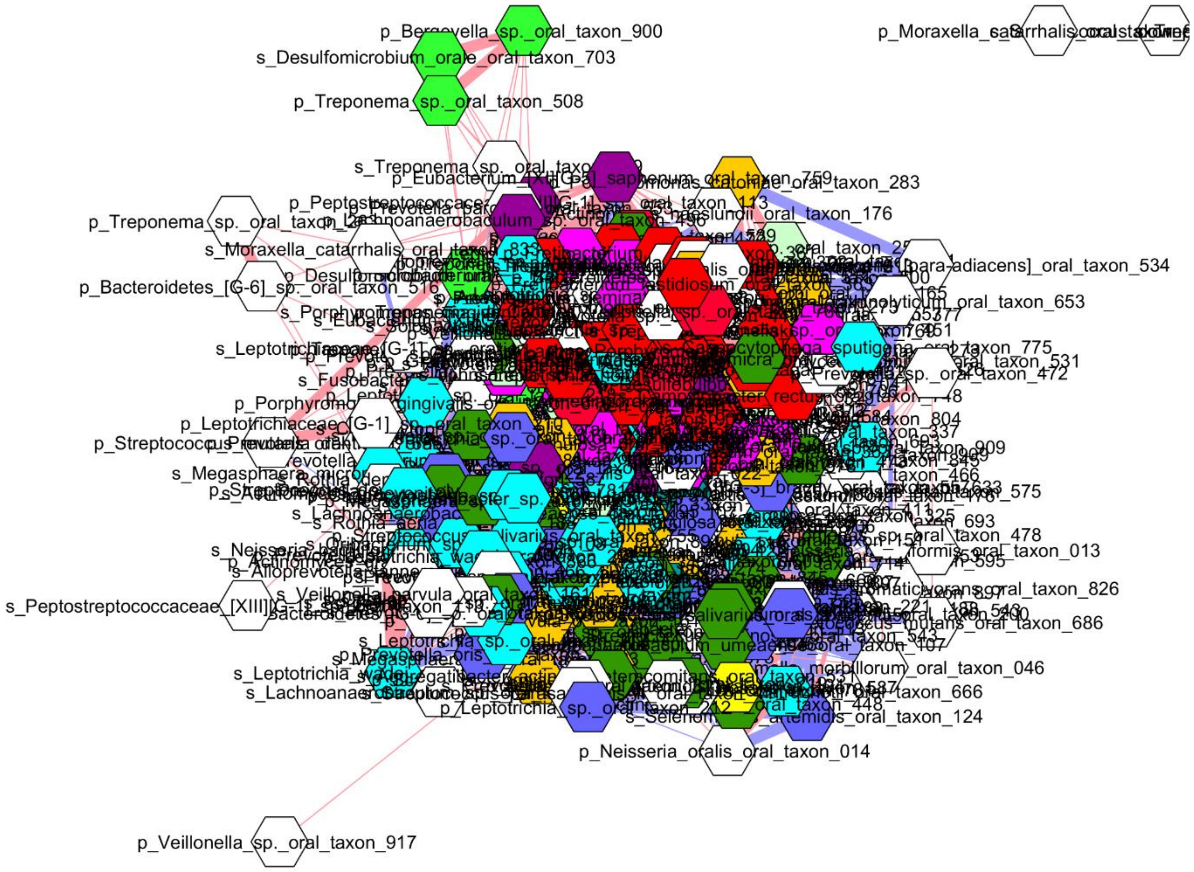

Each node represents a bacterial species and each edge represents a co-occurrence/co-exclusion relationship. Nodes are coloured based on modules which are highly connected. Edge width is proportional to the correlation coefficient and colour indicates the sign of the association (blue: negative, pink: positive).

Fig. S8 Pairwise Spearman's correlation between Gingival Bleeding Index (GBI) and species in subgingival plaque and saliva samples.

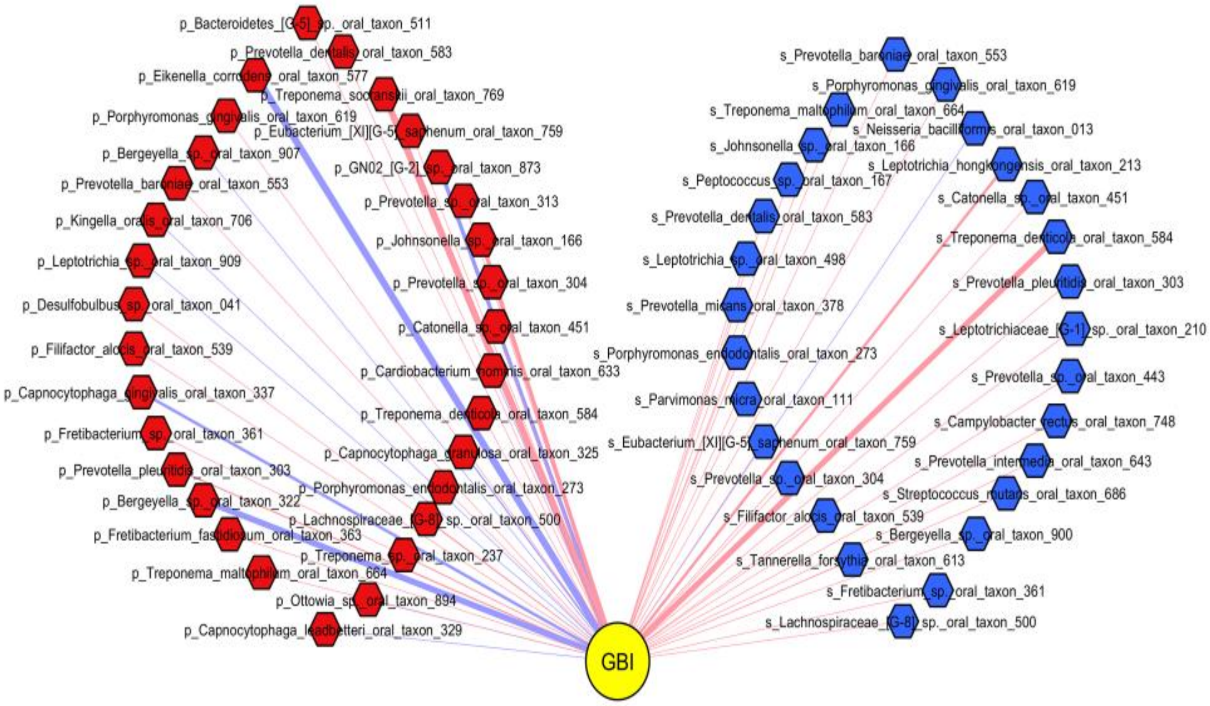

Each node represents a bacterial species and each edge represents a co-occurrence/co-exclusion relationship. Red nodes indicate subgingival plaque species and blue node represents salivary species. Edge width is proportional to the correlation coefficient and colour indicates the sign of the association (blue: negative, pink: positive).
