# Supplementary material for: Keystone Species in Pregnancy Gingivitis: A Snapshot of Oral Microbiome During Pregnancy and Postpartum Period
Source: Front Microbiol. 2018 Oct 9;9:2360. doi: 10.3389/fmicb.2018.02360 (PMC6189292; doi:10.3389/fmicb.2018.02360)
Supplement: Supplementary file 2 [file Data_Sheet_2.docx]

**Table S1** Sociodemographic and oral health characteristics (n = 24)

| Demographic characteristics | Mean (± SD) | N (%) |
| --- | --- | --- |
| Age in years | 31.9 (± 5.4) years |  |
| Gestational age |  |  |
| First trimester | 8.9 (± 3.3) weeks |  |
| Second trimester | 21.7 (± 1.4) weeks |  |
| Third trimester | 31.6 (± 1.5) weeks |  |
| Smoking status |  |  |
| Yes |  | 0 (0.0 %) |
| No |  | 24 (100%) |
| Oral health characteristics | | |
| Brushing frequency during pregnancy |  |  |
| More than twice a day |  | 1 (4.2%) |
| Twice a day |  | 20 (83.3%) |
| Once a day |  | 3 (12.5%) |
| Ainamo Gingival bleeding index |  |  |
| First trimester | 0.13 (± 0.17) |  |
| Second trimester | 0.27 (±0.30) |  |
| Third trimester | 0.12 (±0.22) |  |

Table S2: Results of PERMANOVA analysis of the Bray-Curtis dissimilarities for bacterial OTU to associate microbiome structure with trimesters and patient metadata.


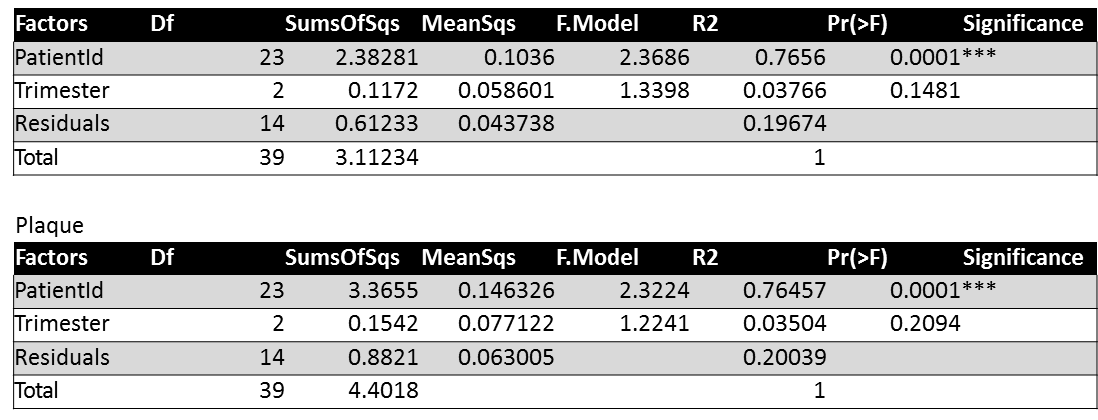


Table S3. Highly connected modules in network based analysis during pregnancy (prefix ‘p’ and‘s’ denote species from subgingival plaque and saliva respectively)

| MCODE App Results | |  |  |  |
| --- | --- | --- | --- | --- |
| Date: 28-Apr-2018 14:57:36 | | |  |  |
|  |  |  |  |  |
| Parameters: | |  |  |  |
| Network Scoring: | |  |  |  |
| Include Loops: false Degree Cutoff: 2 | | | |  |
| Cluster Finding: | |  |  |  |
| Node Score Cutoff: 0.2 Haircut: true Fluff: false K-Core: 2 Max. Depth from Seed: 100 | | | | |
|  |  |  |  |  |
| Cluster | Score (Density*#Nodes) | Nodes | Edges | Node IDs |
| 1 | 20.514 | 36 | 359 | s_Eubacterium_[XI][G5]_saphenum_oral_taxon_759, p_Fretibacterium_sp._oral_taxon_361, p_Fretibacterium_fastidiosum_oral_taxon_363, s_Prevotella_sp._oral_taxon_526, s_Eubacterium_[XI][G-3]_brachy_oral_taxon_557, p_Filifactor_alocis_oral_taxon_539, s_Prevotella_sp._oral_taxon_443, p_Eubacterium_[XI][G5]_saphenum_oral_taxon_759, p_Prevotella_sp._oral_taxon_526, s_Catonella_sp._oral_taxon_451, p_Desulfobulbus_sp._oral_taxon_041, p_Prevotella_sp._oral_taxon_304, s_Prevotella_pleuritidis_oral_taxon_303, s_Campylobacter_rectus_oral_taxon_748, s_Prevotella_intermedia_oral_taxon_643, s_Prevotella_dentalis_oral_taxon_583, s_Prevotella_baroniae_oral_taxon_553, p_Prevotella_intermedia_oral_taxon_643, s_Porphyromonas_gingivalis_oral_taxon_619, s_Porphyromonas_endodontalis_oral_taxon_273, p_Porphyromonas_endodontalis_oral_taxon_273, s_Treponema_sp._oral_taxon_237, s_Treponema_socranskii_oral_taxon_769, s_Treponema_maltophilum_oral_taxon_664, p_Treponema_sp._oral_taxon_258, s_Treponema_denticola_oral_taxon_584, p_Treponema_sp._oral_taxon_237, s_Tannerella_forsythia_oral_taxon_613, p_Treponema_maltophilum_oral_taxon_664, s_Lachnospiraceae_[G-8]_sp._oral_taxon_500, p_Treponema_denticola_oral_taxon_584, p_Lachnospiraceae_[G-8]_sp._oral_taxon_500, p_Johnsonella_sp._oral_taxon_166, s_Fretibacterium_sp._oral_taxon_361, s_Fretibacterium_fastidiosum_oral_taxon_363, s_Filifactor_alocis_oral_taxon_539 |
| 2 | 5.333 | 7 | 16 | s_Prevotella_maculosa_oral_taxon_289, s_Dialister_invisus_oral_taxon_118, s_Veillonellaceae_[G-1]_sp._oral_taxon_155, s_Streptococcus_anginosus_oral_taxon_543, s_Prevotella_sp._oral_taxon_292, s_Alloprevotella_tannerae_oral_taxon_466, s_Prevotella_oris_oral_taxon_311 |
| 3 | 5 | 5 | 10 | s_Treponema_sp._oral_taxon_263, p_Desulfomicrobium_orale_oral_taxon_703, s_Treponema_sp._oral_taxon_239, p_Treponema_sp._oral_taxon_263, s_Streptococcus_downei_oral_taxon_594 |
| 4 | 4.5 | 5 | 9 | p_SR1_[G-1]_sp._oral_taxon_345, p_Ottowia_sp._oral_taxon_894, p_Capnocytophaga_granulosa_oral_taxon_325, p_GN02_[G-2]_sp._oral_taxon_873, p_Bergeyella_sp._oral_taxon_907 |
| 5 | 4 | 4 | 6 | s_Actinomyces_sp._oral_taxon_448, s_Actinomyces_naeslundii_oral_taxon_176, s_Actinobaculum_sp._oral_taxon_183, s_Corynebacterium_matruchotii_oral_taxon_666 |
| 6 | 3.6 | 6 | 9 | p_Prevotella_baroniae_oral_taxon_553, p_Porphyromonas_gingivalis_oral_taxon_619, p_Catonella_sp._oral_taxon_451, s_Parvimonas_micra_oral_taxon_111, s_Johnsonella_sp._oral_taxon_166, s_Leptotrichia_hongkongensis_oral_taxon_213 |
| 7 | 3 | 3 | 3 | s_Oribacterium_sp._oral_taxon_108, s_Selenomonas_sp._oral_taxon_478, s_Leptotrichia_sp._oral_taxon_221 |
| 8 | 3 | 3 | 3 | p_Bacteroidales_[G-2]_sp._oral_taxon_274, p_Capnocytophaga_sp._oral_taxon_326, s_Capnocytophaga_sp._oral_taxon_326 |

Table S4. Highly connected modules in network based analysis during postpartum period (prefix ‘p’ and‘s’ denote species from subgingival plaque and saliva respectively)

| MCODE App Results | | |  |  |  |
| --- | --- | --- | --- | --- | --- |
| Date: 27-Apr-2018 11:50:45 | | | |  |  |
|  | |  |  |  |  |
| Parameters: | | |  |  |  |
| Network Scoring: | | |  |  |  |
| Include Loops: false Degree Cutoff: 2 | | | | |  |
| Cluster Finding: | | |  |  |  |
| Node Score Cutoff: 0.2 Haircut: true Fluff: false K-Core: 2 Max. Depth from Seed: 100 | | | | | |
|  |  | |  |  |  |
| Cluster | Score (Density*#Nodes) | | Nodes | Edges | Node IDs |
| 1 | 40.837 | | 44 | 878 | s_Porphyromonas_endodontalis_oral_taxon_273, p_Bergeyella_sp._oral_taxon_322, p_Porphyromonas_endodontalis_oral_taxon_273, s_Others(<0.5%), p_Alloprevotella_rava_oral_taxon_302, s_Veillonellaceae_[G-1]_sp._oral_taxon_155, s_Veillonella_sp._oral_taxon_780,  p_Veillonellaceae_[G-1]_sp._oral_taxon_155, p_Veillonella_sp._oral_taxon_780, s_Leptotrichia_sp._oral_taxon_498, s_Treponema_sp._oral_taxon_237, s_Treponema_socranskii_oral_taxon_769, p_Treponema_sp._oral_taxon_258, s_Treponema_denticola_oral_taxon_584, s_Leptotrichia_hongkongensis_oral_taxon_213, p_Treponema_sp._oral_taxon_237, s_Tannerella_forsythia_oral_taxon_613, s_Lachnospiraceae_[G-8]_sp._oral_taxon_500, p_Treponema_denticola_oral_taxon_584, p_Leptotrichia_hongkongensis_oral_taxon_213, p_Tannerella_forsythia_oral_taxon_613, s_Johnsonella_sp._oral_taxon_166, p_Lachnospiraceae_[G-8]_sp._oral_taxon_500, p_Johnsonella_sp._oral_taxon_166, s_Fretibacterium_sp._oral_taxon_361, p_Granulicatella_adiacens_[para-adiacens]_oral_taxon_534, s_Fretibacterium_fastidiosum_oral_taxon_363, s_Filifactor_alocis_oral_taxon_539, p_Fretibacterium_sp._oral_taxon_361, s_Prevotella_sp._oral_taxon_526, p_Fretibacterium_fastidiosum_oral_taxon_363, p_Filifactor_alocis_oral_taxon_539, s_Prevotella_sp._oral_taxon_443, s_Desulfobulbus_sp._oral_taxon_041, p_Prevotella_sp._oral_taxon_526, p_Eikenella_corrodens_oral_taxon_577, p_Desulfobulbus_sp._oral_taxon_041, s_Campylobacter_rectus_oral_taxon_748, p_Capnocytophaga_leadbetteri_oral_taxon_329, s_Prevotella_intermedia_oral_taxon_643, s_Prevotella_denticola_oral_taxon_291, s_Bergeyella_sp._oral_taxon_900, p_Prevotella_micans_oral_taxon_378, p_Prevotella_intermedia_oral_taxon_643 |
| 2 | 10.286 | | 36 | 180 | s_Bacteroidales_[G-2]_sp._oral_taxon_274, s_Porphyromonas_gingivalis_oral_taxon_619, s_Alloprevotella_sp._oral_taxon_912, s_Alloprevotella_sp._oral_taxon_473, p_Porphyromonas_gingivalis_oral_taxon_619, s_Ottowia_sp._oral_taxon_894, s_Oribacterium_sp._oral_taxon_108, s_Neisseria_flava_oral_taxon_609, p_Aggregatibacter_sp._oral_taxon_513, s_Actinomyces_graevenitzii_oral_taxon_866, p_Unclassified, s_Megasphaera_micronuciformis_oral_taxon_122, p_Actinomyces_graevenitzii_oral_taxon_866, s_Leptotrichia_wadei_oral_taxon_222, s_Leptotrichia_sp._oral_taxon_909, p_Megasphaera_micronuciformis_oral_taxon_122, p_Leptotrichia_wadei_oral_taxon_222, s_Leptotrichia_sp._oral_taxon_212, s_Lautropia_mirabilis_oral_taxon_022, p_Streptococcus_salivarius_oral_taxon_755, p_Solobacterium_moorei_oral_taxon_678, p_Dialister_invisus_oral_taxon_118, s_Prevotella_sp._oral_taxon_299, s_Prevotella_sp._oral_taxon_292, s_Prevotella_salivae_oral_taxon_307, p_Corynebacterium_matruchotii_oral_taxon_666, s_Capnocytophaga_sputigena_oral_taxon_775, p_Prevotella_sp._oral_taxon_292, s_Prevotella_oulorum_oral_taxon_288, p_Prevotella_salivae_oral_taxon_307, s_Capnocytophaga_granulosa_oral_taxon_325, p_Capnocytophaga_sputigena_oral_taxon_775, p_Prevotella_pallens_oral_taxon_714, p_Capnocytophaga_sp._oral_taxon_338, p_Prevotella_oulorum_oral_taxon_288, p_Prevotella_maculosa_oral_taxon_289 |
| 3 | 6.5 | | 21 | 65 | s_Selenomonas_sputigena_oral_taxon_151, s_Peptostreptococcus_stomatis_oral_taxon_112, p_Bacteroidetes_[G-5]_sp._oral_taxon_511, p_Selenomonas_sputigena_oral_taxon_151, p_Porphyromonas_catoniae_oral_taxon_283, p_Alloprevotella_sp._oral_taxon_912, p_Alloprevotella_sp._oral_taxon_473, p_Scardovia_wiggsiae_oral_taxon_195, p_Eubacterium_[XI][G-7]_yurii_oral_taxon_377, p_Ottowia_sp._oral_taxon_894, s_Prevotella_sp._oral_taxon_317, s_Corynebacterium_matruchotii_oral_taxon_666, s_Catonella_morbi_oral_taxon_165, p_Prevotella_sp._oral_taxon_313, p_Veillonella_parvula_oral_taxon_161, s_Capnocytophaga_gingivalis_oral_taxon_337, s_Terrahaemophilus_aromaticivorans_oral_taxon_826, p_Capnocytophaga_sp._oral_taxon_326, p_Capnocytophaga_granulosa_oral_taxon_325, p_Lautropia_mirabilis_oral_taxon_022, s_Bergeyella_sp._oral_taxon_322 |
| 4 | 5.846 | | 14 | 38 | p_Prevotella_dentalis_oral_taxon_583, s_Anaeroglobus_geminatus_oral_taxon_121, s_Fusobacterium_nucleatum_ss_vincentii_oral_taxon_200, p_Anaeroglobus_geminatus_oral_taxon_121, s_Dialister_invisus_oral_taxon_118, s_Catonella_sp._oral_taxon_451, s_Prevotella_pleuritidis_oral_taxon_303, s_Prevotella_pallens_oral_taxon_714, p_Catonella_sp._oral_taxon_451, p_Treponema_sp._oral_taxon_239, p_Leptotrichia_sp._oral_taxon_498, s_Prevotella_nigrescens_oral_taxon_693, p_Treponema_socranskii_oral_taxon_769, s_Prevotella_micans_oral_taxon_378 |
| 5 | 5.769 | | 27 | 75 | s_Aggregatibacter_actinomycetemcomitans_oral_taxon_531, s_Actinomyces_sp._oral_taxon_448, p_Parvimonas_micra_oral_taxon_111, s_Actinobaculum_sp._oral_taxon_183, p_Actinobaculum_sp._oral_taxon_183, p_Megasphaera_sp._oral_taxon_123, p_Leptotrichia_sp._oral_taxon_221, s_Leptotrichia_goodfellowii_oral_taxon_845, p_Leptotrichia_sp._oral_taxon_219, p_Treponema_lecithinolyticum_oral_taxon_653, s_Streptococcus_sp._oral_taxon_058, s_Streptococcus_salivarius_oral_taxon_755, p_Leptotrichia_goodfellowii_oral_taxon_845, p_Lachnoanaerobaculum_umeaense_oral_taxon_107, s_Selenomonas_sp._oral_taxon_478, s_GN02_[G-2]_sp._oral_taxon_873, s_SR1_[G-1]_sp._oral_taxon_875, p_Gemella_morbillorum_oral_taxon_046, s_SR1_[G-1]_sp._oral_taxon_345, p_GN02_[G-2]_sp._oral_taxon_873, s_Prevotella_sp._oral_taxon_472, p_SR1_[G-1]_sp._oral_taxon_345, s_Prevotella_sp._oral_taxon_313, s_Corynebacterium_durum_oral_taxon_595, p_Prevotella_saccharolytica_oral_taxon_781, s_Prevotella_baroniae_oral_taxon_553, p_Bergeyella_sp._oral_taxon_907 |
| 6 | 4.7 | | 21 | 47 | s_Selenomonas_artemidis_oral_taxon_124, p_Streptococcus_anginosus_oral_taxon_543, s_Rothia_dentocariosa_oral_taxon_587, s_Eubacterium_[XI][G-7]_yurii_oral_taxon_377, p_Fusobacterium_nucleatum_ss_vincentii_oral_taxon_200, s_Rothia_aeria_oral_taxon_188, s_Eubacterium_[XI][G-3]_brachy_oral_taxon_557, s_Eikenella_corrodens_oral_taxon_577, s_Unclassified, p_Oribacterium_sp._oral_taxon_108, s_Cardiobacterium_hominis_oral_taxon_633, p_Corynebacterium_durum_oral_taxon_595, s_Leptotrichia_sp._oral_taxon_219, s_Prevotella_melaninogenica_oral_taxon_469, s_Prevotella_maculosa_oral_taxon_289, p_Leptotrichia_sp._oral_taxon_212, p_Prevotella_oris_oral_taxon_311, s_Lachnoanaerobaculum_umeaense_oral_taxon_107, p_Campylobacter_rectus_oral_taxon_748, p_Prevotella_melaninogenica_oral_taxon_469, s_Streptococcus_anginosus_oral_taxon_543 |
| 7 | 3.6 | | 6 | 9 | p_Bergeyella_sp._oral_taxon_900, p_Desulfomicrobium_orale_oral_taxon_703, p_Treponema_sp._oral_taxon_508, s_Veillonella_sp._oral_taxon_917, s_Desulfomicrobium_orale_oral_taxon_703, s_Prevotella_sp._oral_taxon_304 |
| 8 | 3.333 | | 4 | 5 | p_SR1_[G-1]_sp._oral_taxon_875, p_Porphyromonas_sp._oral_taxon_279, s_Bergeyella_sp._oral_taxon_907, p_Cardiobacterium_hominis_oral_taxon_633 |
| 9 | 3.143 | | 8 | 11 | p_Prevotella_sp._oral_taxon_317, s_Treponema_sp._oral_taxon_258, p_Prevotella_sp._oral_taxon_304, p_Catonella_morbi_oral_taxon_165, s_Capnocytophaga_leadbetteri_oral_taxon_329, p_Capnocytophaga_gingivalis_oral_taxon_337, p_Others(<0.5%), s_Prevotella_dentalis_oral_taxon_583 |
| 10 | 3 | | 3 | 3 | p_Rothia_aeria_oral_taxon_188, s_Parvimonas_micra_oral_taxon_111, p_Rothia_dentocariosa_oral_taxon_587 |
| 11 | 2.8 | | 6 | 7 | p_Peptostreptococcaceae_[XIII][G-1]_sp._oral_taxon_113, p_Lachnoanaerobaculum_sp._oral_taxon_496, p_Treponema_maltophilum_oral_taxon_664, p_Peptococcus_sp._oral_taxon_167, p_Eubacterium_[XI][G-5]_saphenum_oral_taxon_759, s_Peptococcus_sp._oral_taxon_167 |
